# An *nhr-23::mScarlet::3xMyc* knock-in allele for studying spermatogenesis and molting

**DOI:** 10.17912/micropub.biology.000996

**Published:** 2023-10-02

**Authors:** Krista M. Myles, James Matthew Ragle, Jordan D. Ward

**Affiliations:** 1 Department of Molecular, Cell, and Developmental Biology, University of California, Santa Cruz, Santa Cruz, CA 95064

## Abstract

*C. elegans* NHR-23 is a nuclear hormone receptor transcription factor involved in molting, apical extracellular matrix structure, and spermatogenesis. To determine NHR-23 expression dynamics, we previously tagged the endogenous *nhr-23* locus with a GFP::AID*::3xFLAG tag. To allow co-localization of NHR-23 with green fluorescent protein-tagged factors of interest, we generated an equivalent strain carrying an *mScarlet::3xMyc* tag to produce a C-terminal fusion. Similar to the GFP::AID*::3xFLAG knock-in, NHR-23 ::mScarlet::3xMyc was expressed in seam and hypodermal cells, vulval precursor cells, and the spermatogenic germline. We also observed a diffuse NHR-23::mScarlet expression pattern in spermatids and residual bodies after NHR-23 ceased to localize on chromatin. Further examination of this novel localization may provide insight into NHR-23 regulation of spermatogenesis.

**Figure 1. NHR-23 ::mScarlet::3xMyc expression in epithelial and germline cells:**
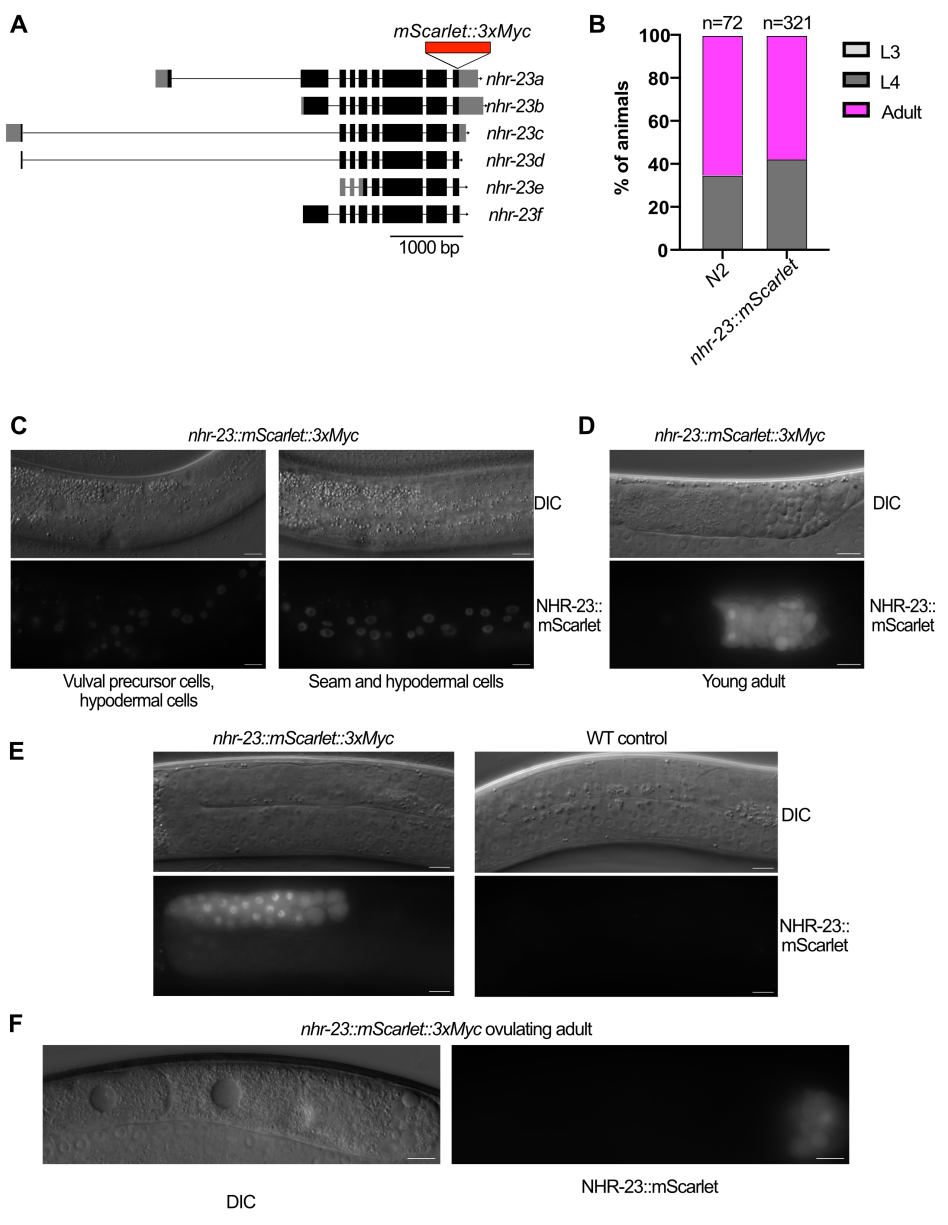
(A) Schematic of the *nhr-23* gene with location of the endogenous *mScarlet::3xMyc* knock-in. Black rectangles are coding exons, gray rectangles are the 5’ and 3’ untranslated regions, and the arrow indicates the direction of the gene and position of the introns. (B) Developmental timing assay. Strains of the indicated genotype were allowed to lay eggs for one hour at 25ºC before removal and the developmental stage of their progeny was scored 50 hours later. Two independent experiments were performed and the number of animals scored is indicated above each bar. (C) Expression of NHR-23 ::mScarlet in L4.2 vulval precursor cells, seam cells, and hypodermal cells. (D-F) Expression of NHR-23::mScarlet::3xMyc in germlines of the indicated developmental stages. A wild-type control is included in (E). Scale bars=10 µm in C-F. All images are representative of twenty animals examined over two independent experiments.

## Description

Nematode molting is emerging as a model to understand the mechanisms underpinning developmentally programmed apical extracellular matrix remodeling and oscillatory gene expression (Cohen and Sundaram 2020; Tsiairis and Großhans 2021). Nuclear Hormone Receptor-23 ( NHR-23 ) is an important regulator of both of these processes as well as spermatogenesis (Kostrouchova et al. 1998, 2001; Kouns et al. 2011; Ragle et al. 2020, 2022; Patel et al. 2022; Johnson et al. 2023; Kinney et al. 2023) . NHR-23 is homologous with the mammalian circadian rhythm regulator RORα and the insect molting regulator DHR3 (Lam et al. 1997; Jetten 2009; Ruaud et al. 2010) . We previously generated a nhr-23 ::GFP::AID*::3xFLAG knock-in to produce a C-terminal fusion that labels all isoforms and allows for conditional NHR-23 depletion using the auxin-inducible degron system (Nishimura et al. 2009; Zhang et al. 2015; Ragle et al. 2020; Ashley *et al.* 2021). To allow for co-localization analysis of NHR-23 with green fluorescent protein-tagged factors we created an equivalent *nhr-23 ::mScarlet::3xMyc* strain using CRISPR/Cas9-mediated genome editing ( Figure 1A ). To test whether the mScarlet::3xMyc tag compromised NHR-23 function, we measured developmental speed and found that *nhr-23 ::mScarlet::3xMyc* animals had a comparable developmental rate to wild-type controls ( Figure 1B ).

The utility of this strain is highlighted by a recent study comparing NHR-23 ::mScarlet::3xMyc expression to that of NHR-85 ::GFP and LIN-42 ::YFP (Kinney et al. 2023) , two other molting regulators (Jeon *et al.* 1999; Gissendanner et al. 2004; Monsalve et al. 2011). This work found that NHR-23 and NHR-85 cooperated to promote the expression of the *lin-4* microRNA at specific points in development, and reported that NHR-23 ::mScarlet::3xMyc peaks in expression between L4.2 and L4.3  (Kinney et al. 2023) . We found a similar pulse of NHR-23 ::mScarlet::3xMyc in L4.2 vulval precursor cells ( Figure 1C ), which is recapitulated in an nhr-23 ::GFP::AID*::3xFLAG strain (Johnson et al. 2023) . NHR-23 ::mScarlet::3xMyc was also detectable in seam and hypodermal cells at this stage, epithelial cells that synthesize cuticular components (Lažetić and Fay 2017)( Figure 1C ). NHR-23 ::mScarlet::3xMyc was detected in pachytene nuclei in L4 animals ( Figure 1E ), and its zone of expression became restricted in young adult animals ( Figure 1D ). This expression pattern is again similar to NHR-23::GFP::AID*::3xFLAG (Ragle et al. 2020) . However, we also observed some interesting differences compared to NHR-23 ::GFP::AID*::3xFLAG. In our previous work, we observed that NHR::23::GFP became undetectable in ovulating adults (Ragle et al. 2020) . In contrast, NHR-23 ::mScarlet::3xMyc appears to be diffusely expressed in young adult residual bodies and spermatocytes ( Figure 1D ). This diffuse expression persists into ovulating adults, and appears restricted to the spermatheca ( Figure 1F ). The NHR-23::mScarlet::3xMyc signal is specific to *nhr-23 ::mScarlet::3xMyc* animals as no diffuse expression in the germline is observed in wild-type animals ( Figure 1E ). One can observe the diffuse expression pattern in L4.5 germlines after NHR-23 ::mScarlet::3xMyc localization is lost from nuclei ( Figure 1E ).

One outstanding question is what might account for these differences in NHR-23 expression patterns in the GFP and mScarlet fusions? One possible explanation is that gut autofluorescence, which is prominent in the GFP excitation/emission range, could be masking diffuse NHR-23 expression. Use of specific filter sets or genetic perturbations to reduce or eliminate autofluorescence will be an important future direction (Hermann et al. 2005; Coburn et al. 2013; Teuscher and Ewald 2018) . While the NHR-23 ::GFP and NHR-23::mScarlet patterns need to be closely compared, the diffuse NHR-23::mScarlet::3xFLAG expression may challenge the apparent rapid removal of NHR-23 from the germline that the NHR-23::GFP expression pattern suggested. The *nhr-23 ::mScarlet::3xMyc* will be a useful reagent to study the role of NHR-23 in regulating gene expression during spermatogenesis, developmental timing, and apical extracellular matrix remodeling.

## Methods

*C. elegans strains and culture*

*C. elegans* strains (see table in Reagents) were cultured as originally described (Brenner 1974) , except worms were grown on MYOB instead of NGM. MYOB was made as previously described (Church et al. 1995) . Animals were cultured at 20°C for all assays, unless otherwise indicated. For general strain propagation, animals were grown at 15°C according to standard protocols. Brood sizes were performed as previously described (Ragle et al. 2022) , except that they were performed at 25ºC to add mild heat-stress to test for cryptic reduction-of-function phenotypes. Developmental timing assays were performed by placing 20 adults of the indicated genotype on 6cm MYOB plates for one hour at 25ºC. The parent animals were removed and plates were incubated for a further 52 hours at 25ºC before scoring for developmental stage.

*Strain generation*

Knock-ins were generated by the self-excising cassette (SEC) CRISPR method (Dickinson et al. 2015) . A *U6p::sgRNA(F+E)* vector (pJW1856) targeting the 3’ end of *nhr-23* was generated by annealing oligos 3488+3489 and SapTrap cloning into pJW1838 (Ashley et al. 2021) , as previously described (Schwartz and Jorgensen 2016) . An *nhr-23 ::30 amino acid (aa) linker::mScarlet-I^SEC^3xMyc* repair template (pJW1877) was assembled through SapTrap cloning with pMLS257 (backbone), pJW1776 ( *nhr-23* 5’ homology arm), pJW1781 ( *nhr-23* 3’ homology arm), and pJW1816 ( *30aa linker::mScarlet-I^SEC^3xMyc* ) (Schwartz and Jorgensen 2016; Ragle et al. 2020; Ashley et al. 2021) . The plasmid was injected into EG9615 (Schwartz et al. 2021) , which stably expresses Cas9, and JDW119 knock-in animals were recovered as described (Dickinson et al. 2015) . The SEC was then excised by heat-shock (Dickinson et al. 2015) to generate JDW129. This strain was outcrossed four times against wild-type N2 animals to remove the Cas9 transgene and the *unc-119 ( ed3 )* allele. The loss of the *oxSi1091* Cas9 transgene was confirmed by genotyping with oligos 5934+5935 (detects unmodified locus) and 5237+5238 (detects Cas9 transgene in locus). Loss of the *unc-119 ( ed3 )* allele was confirmed by phenotyping. The *nhr-23 ::mScarlet::3xMyc* insertion was genotyped with oligos 1586+1587 (detects wild-type allele and homozygous knock-in allele) and oligos 1586+4120 (knock-in specific). Genotyping reactions were performed using a 63ºC annealing temperature.

*Microscopy*

Animals were picked into a 5 µl drop of M9+5 mM levamisole on a 2% agarose pad on a glass slide and secured with a coverslip. Animals were imaged using a Plan-Apochromat 63×/1.4 Oil DIC lens on an AxioImager M2 microscope (Carl Zeiss Microscopy) equipped with a Colibri 7 LED light source and an Axiocam 506 mono camera. We used Fiji software (version: 2.0.0- rc-69/1.52p) to process images (Schindelin et al. 2012) . For the comparison between N2 control animals and *nhr-23 ::mScarlet::3xMyc* animals, we set the exposure conditions to avoid pixel saturation of the brightest sample and kept equivalent exposure for imaging.

## Reagents

**Table:** 

**Strain**	**Genotype**	**Available from**
N2	WT	CGC
EG9615	oxSi1091[Pmex-5::cas9(+ smu-2 introns):: tbb-2 3'UTR unc-119 +; *ttTi5605] II; unc-119( ed3 ) III	Prof. Erik Jorgensen
JDW119	*nhr-23 (wrd31[nhr-23::30aa linker:mScarlet:SEC:3XMyc]) I; ; oxSi1091 [Pmex-5::Cas9( smu-2 introns) unc-119 +] II; unc-119( ed3 ) III *	Prof. Jordan Ward
JDW129	*nhr-23 (wrd33[nhr-23:30aa linker:mScarlet:3XMyc]) I; ; oxSi1091 [Pmex-5::Cas9( smu-2 introns) unc-119 +] II; unc-119( ed3 ) III *	Prof. Jordan Ward
JDW684	*nhr-23 (wrd33[nhr-23::30aa linker:mScarlet:SEC:3XMyc]) I *	Prof. Jordan Ward

**Table:** 

**Plasmid**	**Reference**	**Notes**	**How to obtain plasmid**
pMLS257	Schwartz et al., 2016	SapTrap destination vector for building repair template only vectors; no sgRNA	Addgene
pJW1816	Ashley et al., 2021	*30aa linker::mScarlet^SEC (Lox511I)^3xMyc* for SapTrap.	Addgene
pJW1877	This study	*nhr-23 ::30 aa linker::mScarlet^SEC+Lox511I^3xMyc* repair template. Assembled by SapTrap using pMLS257 backbone and pJW1816	Prof. Jordan Ward
pJW1856	This study	*nhr-23* 3' end sgRNA(F+E) vector	Prof. Jordan Ward
pJW1838	Ashley et al., 2021	SapTrap sgRNA (F+E) vector, K09B11.2 U6 promoter and 3'UTR	Prof. Jordan Ward
pJW1776	Ragle et al., 2020	*nhr-23* 5' homology arm for Sap Trap (Sap sites in arm are mutated)	Prof. Jordan Ward
pJW1781	Ragle et al., 2020	*nhr-23* 3' homology arm for Sap Trap	Prof. Jordan Ward

**Table:** 

**Oligo number**	**Sequence (5' to 3')**	**Purpose**
1586	GTGTGCGGTGAAAGGTATTCTG	*nhr-23* knock-in genotyping
1587	AATGAGGAACTCTCCTGCAAC	*nhr-23* knock-in genotyping
3488	TTGAGAGCTATTCACTGCAGAT	*nhr-23* 3' end sgRNA
3489	AACATCTGCAGTGAATAGCTCT	*nhr-23* 3' end sgRNA
4120	TGCTTCCTTCCATGTGAACCTTG	*nhr-23* knock-in genotyping
5234	ACGGATGCCTAGTTGCATTGA	Cas9 transgene genotyping
5235	GGCTTGTAACGCGGAATCAC	Cas9 transgene genotyping
5237	CTCGAGAAGATGGACGGAAC	Cas9 transgene genotyping
5238	CATTCCCTCGGTGACGTACT	Cas9 transgene genotyping
